# Analytical performance evaluation of the Helibac rapid urease test for detection of *Helicobacter pylori* urease activity under controlled laboratory conditions

**DOI:** 10.3389/fmicb.2026.1889913

**Published:** 2026-07-28

**Authors:** Anna Siwiec, Agnieszka Siwiec

**Affiliations:** 1 Medical College of Rzeszów University, Rzeszów, Poland; 2 Faculty of Medicine, Medical University of Gdańsk, Gdańsk, Poland

**Keywords:** analytical performance, gastritis, *Helicobacter pylori*, culture, rapid urease test

## Abstract

*Helicobacter pylori* infection remains one of the most prevalent chronic bacterial infections worldwide and is strongly associated with chronic gastritis, peptic ulcer disease, mucosa-associated lymphoid tissue lymphoma, and gastric carcinoma. Accurate diagnosis of infection is essential for successful therapy. Currently available diagnostic methods include both invasive and non-invasive approaches; however, none guarantees complete elimination of false-negative or false-positive results. This study aimed to evaluate the analytical performance of a Polish dry rapid urease test, the Helibac RUT for the detection of *H. pylori* infection and compare it with other commercially available urease tests. Reaction kinetics and detection thresholds were assessed using standardized bacterial suspensions and controlled experimental conditions applied to the Helibac RUT, CLO test, BIOHIT, and ENDOSC-Hp tests. The Helibac RUT also was compared with two other rapid urease tests manufactured in Poland. The evaluated test demonstrated lower analytical detection thresholds and faster reaction kinetics under controlled laboratory conditions compared with the comparator rapid urease tests. The results indicate promising analytical performance characteristics of the Helibac RUT system, including rapid color development and low analytical detection thresholds. These findings support further clinical performance studies using gastric biopsy specimens.

## Introduction

*Helicobacter pylori* infection remains one of the most common chronic bacterial infections in humans. At the same time, its prevalence in the global population has been decreasing slightly but systematically over recent decades (Li et al., 2023). Incidence of infection is not distributed evenly geographically or socially (Matysiak-Budnik and Mégraud, 1997; Venneman et al., 2018; Li et al., 2023). In Europe, it is generally accepted that the lowest infection rates are found in Northern Europe, while the highest rates occur in Eastern and Southern Europe (Venneman et al., 2018). Recent epidemiological studies indicate that Poland remains a country with a relatively high prevalence of *H. pylori* infection compared with many Western European countries despite a gradual decline observed over recent decades (Venneman et al., 2018; Ansari and Yamaoka, 2022; Li et al., 2023). Several factors have been proposed to contribute to this phenomenon, including historical cohort effects, socioeconomic disparities, household crowding during childhood, intrafamilial transmission, and regional differences in sanitation standards. Moreover, infection prevalence remains substantially higher among older generations, reflecting acquisition of infection during periods characterized by less favorable hygienic and socioeconomic conditions. These factors contribute to the persistence of *H. pylori* infection as a relevant public health issue in Poland (Laszewicz et al., 2014; Uotani and Graham, 2015; Ansari and Yamaoka, 2022).

*H. pylori* infection is associated with a several-fold higher risk of gastric and duodenal ulcer disease (Kuipers et al., 1995). Literature generally accepts that it is the most important, although not the only, risk factor for this disease (Almadi et al., 2024). Furthermore, *H. pylori* infection is strongly associated with the development of gastric cancer, particularly non-intestinal-type adenocarcinoma (Huang et al., 1998). Consequently, estimates indicate that *H. pylori* may be the most significant infectious agent in oncogenesis, more important than Human Papillomavirus and others (de Martel et al., 2020).

However, diagnosis of the infection has direct therapeutic implications (Lehours and Mégraud, 2005). Biopsy-based diagnosis allows correlation of infection diagnosis with assessment of macroscopic and histological changes in the gastric mucosa. In symptomatic patients, endoscopy also enables evaluation of peptic ulcer disease, inflammation, premalignant lesions, or other pathologies of the upper gastrointestinal tract, while the collected biopsy specimen may be used for both histological and microbiological analysis (Rune, 1996; Ricci et al., 2007; Kainz et al., 2021).

Use of RUTs for this purpose is based on a distinctive biological feature of *H. pylori*, namely its high bacterial urease activity. The enzyme catalyzes urea hydrolysis with ammonia production, resulting in alkalinization of the medium by ammonium ions, which can be detected by a color change of pH indicators. Accordingly, RUTs provide functional diagnostics aligned with the pathophysiology of *H. pylori* infection and can be applied immediately after biopsy sampling, without the need for prior culture (Vaira et al., 2000).

However, the sensitivity of RUTs may be reduced in patients with low bacterial density, patchy mucosal colonization, upper gastrointestinal bleeding, or prior treatment with proton pump inhibitors, antibiotics, or bismuth compounds. In bleeding peptic ulcer disease, RUTs maintain high specificity, whereas their sensitivity is reduced. These limitations indicate the need to optimize test design and reaction conditions and to interpret results cautiously in selected patient populations (Gisbert and Abraira, 2006).

Several technical formats are currently used, including gel-based media or slides, test strips or cards, dry paper matrices, liquid tube-based tests, and cartridges or cassettes with gel wells (Table 1). In comparative studies, CLO test, Hpfast, PyloriTek, Pronto Dry, Pyloriset Urease, and liquid or home-use tests are most frequently reported; in routine clinical practice, Endosc-HP and HUT-test are also used (Laine et al., 1996; Kuo et al., 2002; Dechant et al., 2020; Parihar and McNamara, 2021).

The speed and quality of the test signal depend on the efficiency of the enzymatic reaction, diffusion of reaction products, characteristics of the reaction medium, and conditions of result interpretation (Urgessa et al., 2023).

An important direction in RUT development has been to reduce the time to result, given that rapid intra-procedural or early post-procedural interpretation is more compatible with biopsy-based endoscopic diagnosis than assays requiring prolonged incubation (Siwiec, 2011).

The cassette configuration may contribute to improved analytical sensitivity and shorter time to result, primarily by enabling rapid diffusion of ammonia to the phenol red-containing layer and reducing the risk of contamination (Ruiz-Haas and Ingle, 2009; Al-Adhami et al., 2016; Dong et al., 2021).

The above provides the rationale for evaluating a structural and matrix-based modification of previous RUT formats, consisting of a closable cassette with mechanical compression of the sample, a layered reaction system with separated urea-containing and indicator components enabling rapid diffusion of ammonia to the phenol red-containing layer. This architecture was implemented in a cassette-based RUT intended for qualitative detection of *H. pylori* urease activity, filed as patent application PL446438A1, under the trade name the Helibac RUT (Siwiec, 2025).

The research problem concerns the analytical diagnostic performance of cassette-based Helibac RUT in a laboratory model of *H. pylori* urease activity detection. Methodologically, the problem involves evaluation of the adopted direction of diagnostic test development, namely whether incorporation of a closable cassette and mechanical sample compression improves organization of the RUT reaction environment. From an applied perspective, the problem concerns the suitability of the Helibac RUT for future clinical evaluation using endoscopic biopsy specimens.

The research question is “does the cassette-based Helibac RUT demonstrate higher analytical diagnostic performance than the comparator RUTs?” The corresponding research hypothesis is “the cassette-based Helibac RUT demonstrates higher analytical diagnostic performance than the comparator RUTs.”

The primary objective of this study was to evaluate the analytical performance of the Helibac RUT rapid urease test under standardized laboratory conditions, including analytical detection threshold, reaction kinetics, and response to predefined urease activity. The study was designed as a preclinical verification step preceding clinical performance evaluation.

## Study aim and objectives

The aim of this study was to evaluate the analytical performance of the Helibac RUT under standardized laboratory conditions. The specific objectives were to determine the bacterial and enzymatic detection thresholds, assess reaction kinetics, compare the performance of the assay with commercially available rapid urease tests, and evaluate the suitability of the cassette-based design for further clinical validation studies.

## Material and methods

### Diagnostic test

The test uses a biological property of *H. pylori*, namely production of urease, which hydrolyzes urea to ammonia and carbon dioxide. The generated ammonia alkalinizes the reaction environment, resulting in a color change of the pH indicator. In the applied design, the indicator is phenol red, and a color change from yellow to pink, amaranth, or crimson constitutes the basis for qualitative interpretation of a positive result (Kostelnik et al., 2016).

The test is configured as a rectangular, tightly closable cassette composed of an upper and a lower part. Both parts are connected along their shorter sides by a flexible tether functioning as a hinge. On the side opposite the hinge, snap-fit elements enable closure of the cassette. The latch structure consists of a protrusion on the upper part and a corresponding recess in the protrusion of the lower part. After closure, both parts of the cassette are therefore mechanically stabilized relative to each other, with no possibility of displacement.

The lower part of the cassette contains a circular reaction well. Above it, on the inner surface of the upper part, there is a circular protrusion terminating in a compression ring. After cassette closure, this ring is positioned above the well and compresses the test material placed inside it. The function of this element is to mechanically stabilize the sample and enable tissue fluid from the biopsy specimen to penetrate the urea substrate.

The Helibac RUT reaction system has a layered architecture. It consists of a urea-containing element, an indicator element, and an openwork plastic mesh separating the two elements. The urea-containing element is made of a filter paper disk impregnated with a solution of urea in demineralized water, then dried and cut into disks under strictly controlled conditions preventing contamination with urease-positive bacteria or alkaline chemical substances. The indicator element is made of filter paper impregnated with phenol red solution. The openwork mesh functions as a separator between these layers while enabling rapid diffusion of gaseous ammonia from the urea-containing layer to the indicator layer.

The test material is placed in the well between the layers. After cassette closure, the sample is compressed by the ring of the circular protrusion of the upper part. If *H. pylori* is present in the material, bacterial urease hydrolyzes the urea contained in the urea-containing element. The generated ammonia diffuses through the openwork mesh into the indicator element, where it increases pH and changes the color of phenol red.

The Helibac RUT test result is qualitative. Absence of color change indicates a negative result, whereas development of a pink, amaranth, or crimson color indicates a positive urease reaction. In analytical testing, reaction intensity may be classified using a semiquantitative scale.

### Research material

The study was designed as an analytical pre-validation assessment preceding clinical validation. Test performance was evaluated *in vitro* using two controlled input systems: defined numbers of *H. pylori* cells and predefined urease activity. This approach was used to assess the intrinsic performance of the test matrix independently of the biological and sampling variability associated with gastric biopsy specimens.

Cultured *H. pylori* strains were used as the biological input material because RUT is a functional assay in which signal generation depends on bacterial urease activity. Standardized bacterial suspensions were used instead of fresh gastric biopsy specimens to enable controlled assessment of the bacterial detection threshold. In biopsy material, bacterial load is not directly controlled, and the RUT result may depend on specimen size and bacterial density within the individual tissue fragment (Laine et al., 1996; Yousfi et al., 1996). Standardization to 0.5 McFarland followed by serial dilution enabled assessment of the relationship between bacterial cell number and the time course and intensity of the color reaction (Mahesh et al., 2025), without interference from biopsy sampling variability.

Purified urease was included as a separate enzymatic input material to assess the response threshold of the chemical-matrix system independently of culture-related biological variables. Since RUT is based on urease-mediated urea hydrolysis followed by alkalinization of the reaction medium, predefined urease units allowed direct determination of the minimum enzymatic activity required to generate a visible or complete color reaction (Laine et al., 1996; Yousfi et al., 1996).

Comparator tests from the same diagnostic category were included to determine whether the evaluated cassette-based system provided more favorable analytical performance than established RUT formats (Laine et al., 1996; Yousfi et al., 1996).

Biological material consisted of wild-type *H. pylori* strains recovered from gastric mucosal biopsy specimens from patients with confirmed infection and isolated by the Microbiology Laboratory of the University of Rzeszów. Colonies harvested from culture plates were suspended in sterile physiological saline, and homogenized. Suspension density was standardized to 0.5 McFarland (corresponding to ca. 10^8^ CFU/mL). Serial dilutions were prepared from the initial suspension to obtain samples containing from 10^6^ to 10^3^ bacterial cells per sample (Jorgensen and Ferraro, 2009).

The enzymatic material consisted of urease applied to the evaluated tests at predefined activity levels. In the main enzymatic assessment, the tested range was 0.10–0.0015 U/test and included the following activities: 0.10, 0.05, 0.025, 0.012, 0.006, 0.003, and 0.0015 U/test. In an additional comparison of selected tests manufactured in Poland, urease activities of 0.006, 0.003, and 0.0015 U were applied in 5 μL of distilled water.

The comparator material consisted of commercial RUTs used for detection of *H. pylori* infection, including CLO test, BIOHIT urease test, and Endosc HP test. Selected rapid urease tests manufactured in Poland were additionally included and evaluated in a comparative system using predefined urease activities.

### Research procedure

This study was designed as an analytical laboratory evaluation conducted in accordance with IVDR (EU 2017/746) principles for performance evaluation of *in vitro* diagnostic devices (European Parliament and Council of the European Union, 2017).

In the bacterial component of the assessment, 20 μL of the prepared *H. pylori* suspension was applied to each test matrix. All analytical experiments were conducted at 21 °C, corresponding to the intended conditions of use of the device in routine endoscopic practice.

For the supplementary comparison of rapid urease tests manufactured in Poland, five replicates were performed for each experimental condition. The time to a positive color reaction and the lowest bacterial cell number yielding a positive result were recorded.

In the enzymatic component, predefined urease activities ranging from 0.10 to 0.0015 U/test were applied to the evaluated tests. Reaction readout was performed after 1, 5, 10, 30, and 60 min. At each time point, the presence and intensity of color change were assessed to determine the relationship between enzymatic activity, reaction rate, and color-readout intensity.

In the additional comparison of tests manufactured in Poland, predefined urease activities were applied in 5 μL of distilled water. The reaction was assessed at 0.006, 0.003, and 0.0015 U, with readouts performed after 5, 15, 30, and 60 min.

Phenol red does not undergo an abrupt transition from yellow to red. With increasing pH and additional redox reactions, it produces a continuum of color shades, with gradual spectral changes around 560 nm (greenish yellow). Although phenol red solution shade is commonly interpreted using a colorimetric curve and therefore as a quantitative variable, this approach would not be preferable in case of biological material. The indicator readily undergoes oxidation and reduction reactions, which may shift the spectral peak through generation of mono- and dihalogenated forms with more intense coloration (Morgan et al., 2019).

**FIGURE 1:**
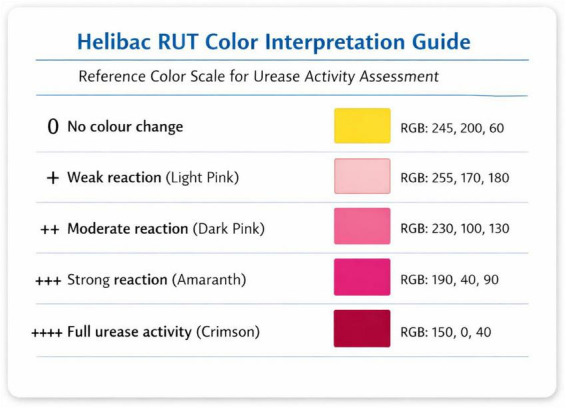
Representative examples of reaction intensity categories used for semiquantitative interpretation of Helibac RUT results.

Accordingly, in biological material, phenol red may show greater color intensity than in a purely chemical matrix, while also showing a more complex relationship between pH and observed color. For this reason, use of an ordinal variable is justified. Such a variable does not assume a simple functional relationship, as would be implied by a ratio-scale variable, but provides more information than a binary variable. In medical diagnostics, ordinal scales are commonly expressed as 0, +, ++, +++, or higher-grade positive categories, depending on the intensity of assessed phenomenon (Delanghe et al., 2017). Representative examples of reaction intensity categories are presented in Figure 1. Color interpretation was performed independently by two trained observers who were blinded to the applied bacterial concentration and urease activity. In case of disagreement, consensus evaluation was performed.

The result was assessed qualitatively based on the intensity of the color change. No color change was classified as a negative result. Positive reactions were recorded using an ordinal scale of increasing intensity: “+” for light pink, “++” for dark pink, “+++” for amaranth, and “++++” for crimson. The full positive reaction endpoint was defined as achievement of the highest color intensity in the adopted readout scale.

In the bacterial suspension assay, the limit of detection was defined as the lowest number of *H. pylori* cells producing a positive readout within the adopted observation period (IJzerman-Boon and van den Heuvel, 2015). In the urease assay, the reaction threshold was defined as the lowest enzyme activity producing a visible positive color change, whereas the full endpoint was defined as the lowest activity leading to a “++++” reaction during the observation period.

Statistical analysis was performed using one-way ANOVA followed by Tukey’s multiple comparison test. Effect size was estimated using η^2^ (eta squared). Ninety-five percent confidence intervals (95% CI) were calculated where applicable. Statistical significance was defined as *p* < 0.05.

The other RUTs used as reference tests were applied in the standard manner, according to the manufacturers’ instructions.

## Results

At the one-minute time point, a positive reaction was observed only for the Helibac RUT test at bacterial loads of 1.0 × 10^7^, 5.0 × 10^6^, and 2.5 × 10^6^ cells, whereas the other tests did not reach any detection threshold within this incubation time (Table 2).

After 60 min, the detection threshold was approx. 3.7 × 10^4^ cells for the Helibac RUT, approximately 6.0 × 10^5^ cells for the CLO test, approx. 6.0 × 10^5^ cells for BIOHIT, and approximately 6.0 × 10^5^ cells for ENDOSC-Hp. This also represented the final detection threshold (Table 2).

Analysis of reaction kinetics demonstrated substantial differences among the evaluated rapid urease tests. The mean time to first positive reaction was shortest for Helibac RUT (17.9 min; 95% CI: 16.42–19.38), followed by CLO test (39.0 min; 95% CI: 35.73–42.27), BIOHIT (85.6 min; 95% CI: 78.78–92.42), and ENDOSC-Hp (91.1 min; 95% CI: 83.57–98.63). The narrow confidence interval observed for Helibac RUT indicates a highly consistent analytical response under standardized laboratory conditions (Table 5).

One-way ANOVA demonstrated significant differences in reaction kinetics among the evaluated rapid urease tests (F(3,32) = 4.20, *p* = 0.013, η^2^ = 0.283). *Post hoc* Tukey analysis demonstrated significantly faster reaction development for the Helibac RUT compared with BIOHIT (adjusted *p* = 0.028) and ENDOSC-Hp (adjusted *p* = 0.019), while the difference versus CLO test did not reach statistical significance (adjusted *p* = 0.086).

Analysis of the urease reaction kinetics demonstrated significant differences among the evaluated tests in both the rate of reaction development and the limit of microbial detection. The Helibac RUT test was characterized by an immediate or very early intense color change at high bacterial loads, with maximum reaction intensity maintained at subsequent time points. As the number of *H. pylori* cells decreased, the time required to obtain a positive reaction gradually increased; however, a positive result remained achievable even at the lowest analyzed concentrations, indicating high analytical sensitivity and a low detection threshold. In contrast, the CLO test showed delayed reaction kinetics, with no response at the earliest time points and loss of reactivity at lower bacterial concentrations. BIOHIT and ENDOSC-Hp showed even more limited reaction dynamics, with positive results only at high bacterial loads and no reaction at low microorganism concentrations (Table 2).

In assays using Sigma urease, the detection threshold at the 1-min time point was 0.012 U of urease for the Helibac RUT; no reaction was observed at 0.003 or 0.0015 U. CLO test, BIOHIT, and ENDOSC-Hp showed no reaction at any of the analyzed enzymatic activities during the first minute of incubation (Table 3).

After 60 min, the detection threshold was 0.0015 U of urease for the Helibac RUT and 0.006 U for CLO test. BIOHIT and ENDOSC-Hp showed no reaction across the entire analyzed range of enzymatic activity, including at 0.1 U.

The final detection threshold, defined as the lowest urease dose producing a positive reaction at any analyzed time point, was 0.0015 U for the Helibac RUT and 0.006 U for CLO test. For BIOHIT and ENDOSC-Hp, the detection threshold was not reached within the tested range of enzymatic activity.

Assessment with standardized reference urease enabled controlled analysis of enzymatic sensitivity across the evaluated diagnostic systems. The Helibac RUT showed a rapid and intense response even at low enzyme doses, with progressive increase in reaction intensity during incubation. This indicates high reactivity of the detection system and efficient ammonia diffusion within the test matrix. CLO test reacted only at higher enzymatic activity, with a delayed and less intense response. BIOHIT and ENDOSC-Hp showed no reaction within the analyzed range of enzymatic activity, indicating markedly lower analytical sensitivity under standardized conditions (Table 3).

Comparison of the three tests manufactured in Poland provided supplementary data to the analyses performed with wild-type bacterial strains and reference urease. The comparison showed a faster reaction kinetics of the Helibac RUT in terms of reaction intensity and reaction rate at the same amount of urease applied to the test matrix. The Helibac RUT produced clearly positive results after shorter incubation and at lower enzymatic activity, whereas the other tests showed weaker, delayed, or absent reactions at lower enzyme doses.

Prolongation of incubation time increased the proportion of positive results in the reference tests; however, reaction intensity remained lower than for the Helibac RUT. This finding suggests a potential structural advantage and improved analytical sensitivity of this diagnostic system compared with the other Polish RUTs (Table 4). A *post hoc* power analysis indicated statistical power exceeding 80% for the primary comparisons of reaction kinetics between the Helibac RUT and comparator assays.

## Discussion

The study hypothesis that the Helibac RUT would demonstrate superior analytical performance compared with established rapid urease tests was supported by the experimental findings. Under standardized laboratory conditions, the Helibac RUT demonstrated lower bacterial detection thresholds, lower enzymatic detection thresholds, and faster reaction kinetics than the evaluated comparator assays. These findings suggest that optimization of the reaction environment may substantially influence analytical performance of rapid urease tests beyond the urease-based detection principle itself (Laine et al., 1996; Niv et al., 1998; Vaira et al., 2010).

For dilutions tested, the Helibac RUT achieved a final detection threshold of approx. 3.7 × 10^4^ *H. pylori* cells after 60 min, whereas reference tests reached threshold of approx. 6.0 × 10^5^ cells. Favorable assessment of the results obtained in this study derives not only from direct comparisons but also from data reported in the literature, which indicate that, for classical RUTs, the detection threshold is close to 10^5^ bacteria in the sample (Uotani and Graham, 2015; Dawod, 2022).

The Helibac RUT produced a positive result as early as 1 min at loads of 1.0 × 10^7^, 5.0 × 10^6^, and 2.5 × 10^6^ cells and, under the same conditions, reached a complete reaction (++++). At lower loads, a positive reaction appeared later, but was still achieved within the observation period. Consequently, the Helibac RUT not only detected lower concentrations of bacterial material than the tests used for comparative purposes, but also generated a readable signal more rapidly at high and intermediate loads (Laine et al., 1996; Niv et al., 1998; Morio et al., 2004; Perna et al., 2005; Vaira et al., 2010; Uotani and Graham, 2015; Dechant et al., 2020).

Considering that diagnosis of infection using RUT does not require enzymatic digestion of tissue material but is based on tissue fluid, it is highly probable that, in clinical applications, the Helibac RUT may be characterized by a particularly rapid time to result. Against this background, there is a substantial advance over older, “classical” tests, including Pyloritek, CLO test, and Hpfast, which in a clinical study achieved median times to a positive result of approx. 30–45 min, while the means for CLO test and Hpfast exceeded 2 h (Laine et al., 1996). Until clinical studies have been conducted, it is not possible to state with certainty that there is a clinical advantage over available dry RUTs, in which times to result are usually shorter than in the oldest RUTs (Kuipers et al., 1995; Yousfi et al., 1996; Laszewicz et al., 2014). It should be noted, however, that Pronto Dry, evaluated against a liquid-phase test, achieved, in a group of untreated patients, a sensitivity of 45% after 5 min, 71.2% after 15 min, 81.1% after 30 min, 90.1% after 3 h, and 91.9% after 24 h, each time outperforming liquid-phase tests (Perna et al., 2005). In another evaluation, sensitivity was 62.5% after 5 min and 84.4% after 30 min, with a specificity of 98.4%, and 75% of positive tests were already positive after 5 min (Morio et al., 2004). In the case of the Helibac RUT, the observed analytical characteristics suggest the potential for favorable performance in future clinical studies. Since individual tests are based on the same enzymatic mechanism, they generally show similarly high specificity, typically ranging from 99% to 100%. However, they differ in overall sensitivity and in sensitivity at early readout. For CLO test, Hpfast, and Pyloritek, the reported overall sensitivities are 93%, 88%, and 89%, respectively, whereas the corresponding sensitivities at rapid readout are 71%, 66%, and 89% (Laine et al., 1996; Dechant et al., 2020).

A potential limitation is that experiments were performed at a single ambient temperature (21 °C). However, this temperature reflects the intended use environment of the device and therefore provides a clinically relevant analytical assessment.

The literature on RUT suggests that the clinical utility of these tests depends not only on chemical reactivity itself, but also on how the test performs on biopsy specimens. In practice, biopsy site, number of specimens collected, uneven distribution of *H. pylori* in the mucosa, prior use of proton pump inhibitors, antibiotics or bismuth, intestinal metaplasia, and other factors are relevant. Further clinical studies should take into account the suggestions from the literature and the need to control factors responsible for false-negative results (Uotani and Graham, 2015; Park and Shin, 2022). Until these studies are conducted, there is a risk of confounding factors that may prevent the favorable *in vitro* results from being translated into clinical trials. In particular, it should be assumed that the modified reaction environment in the Helibac RUT test design may reduce the risk of contamination (Ruiz-Haas and Ingle, 2009; Dong et al., 2021) but also accelerate the dispersion of substances that expose methyl red to oxidation. The risk is the tendency of methyl red to have a higher color expression in the oxidation-reduction environment of biological material (Morgan et al., 2019), which could have a positive effect on diagnostic thresholds, but also lead to false positive or ambiguous results.

In a study by Niv et al. (1998) comparing HUT with PUT, PUT produced a positive result within 10 min in 55% of positive cases, compared with 10% for HUT. Both methods showed a specificity of 97%, while PUT showed higher sensitivity than HUT: 90.3% versus 80.7%, respectively (Laine et al., 1996; Yousfi et al., 1996; Dechant et al., 2020; Dawod, 2022). These findings indicate that the urease-based principle alone does not determine assay rapidity; the technical configuration of the reaction system is a major determinant of time to result (Niv et al., 1998; Nguyen et al., 1999).

At the current stage of research, the changed PUT design in the Helibac RUT has resulted in better analytical efficiency. The higher analytical efficiency of the Helibac RUT compared with the tested comparators may be interpreted as a direct consequence of the configuration of the reaction environment of the layered assay format within a closed cassette with mechanical compression. This assumption is reflected by the high sensitivity to urease activity and the rapid reaction of the test upon exposure to Sigma urease. The Helibac RUT reached a detection threshold of 0.012 U after 1 min and, after 60 min, showed a positive reaction at 0.0015 U. The final enzymatic detection threshold was therefore 0.0015 U, whereas CLO test reached a threshold of 0.006 U, and BIOHIT and ENDOSC-Hp showed no reaction within the analyzed range of enzymatic activity. This finding clearly distinguishes the intrinsic performance of the reaction matrix from variability attributable to biological material. In the context of further RUT development, it may be interpreted as empirical confirmation of the validity of the adopted design approach. However, caution is necessary as the study does not yet demonstrate which exact feature of the cassette is responsible for the observed effect. The closable cassette, mechanical compression, layered separation of the urea and indicator elements, and short ammonia diffusion path operate here as an integrated system. The obtained results indicate only that this system organizes the reaction environment more effectively than the comparator solutions, but they do not encompass all possible configurations aimed at shortening the ammonia diffusion path and do not allow for an individual assessment of the respective structural components.

When considering the clinical implications of the obtained results, it should be borne in mind that the contemporary standard of *H. pylori* diagnostics includes invasive, endoscopic, and non-invasive methods (Vaira et al., 2000; Ricci et al., 2007; Urgessa et al., 2023). There is no single standard diagnostic method; rather, the choice depends on the patient’s medical history and other clinical factors (Urgessa et al., 2023). RUTs are not the “gold standard” in the diagnosis of *H. pylori* infection. The literature recommends the use of at least two different diagnostic methods (Urgessa et al., 2023). The obtained results should therefore not be interpreted as yielding a universal diagnostic tool free from limitations. On the other hand, the results obtained in comparative studies, which were superior in every category to all other tested assays, suggest that the Helibac RUT represents a promising candidate assay with favorable analytical performance characteristics.

The Helibac RUT, like other RUTs, will not replace comprehensive microbiological and histological diagnostics, especially molecular diagnostic methods, relative to which it constitutes a complementary tool enabling rapid confirmation of a diagnosis established primarily on the basis of clinical data. At present, RUT, even despite very high analytical performance parameters, cannot constitute the final link in the diagnostic process, since, in view of the increasing prevalence of *H. pylori* resistance, including to metronidazole, levofloxacin, and clarithromycin, empirical therapy cannot be recommended (Laszewicz et al., 2014).

This results primarily from the systematic increase in the importance of *H. pylori* antibiotic resistance, and thus from the need to determine antimicrobial susceptibility and to assess eradication success (Ansari and Yamaoka, 2022). The increasing importance of antibiotic resistance, combined with the expanding use of molecular diagnostic methods, indicates the need to search for biomarkers, including genotypic biomarkers, of antibiotic resistance and eradication efficacy (Kuipers et al., 1995; Laszewicz et al., 2014; Ansari and Yamaoka, 2022), which, at the present stage of scientific knowledge, cannot be provided by RUTs or similar rapid diagnostic methods.

The results should be considered in the context of the diagnostic functions of RUTs. It is possible that the Helibac RUT, while displaying features of a potentially highly sensitive analytical assay may not affect clinical or diagnostic decision-making directly. The high specificity of RUTs, with sensitivity limited by the specific type of the diagnostic material, means that these are typical tests for confirming infection. This limits RUTs in terms of assessing the effectiveness of therapy. Positive biopsy RUT generally confirms persistent infection (that is eradication therapy failure), but negative result without confirmation by another method cannot at present be regarded as definitive evidence of eradication (Almadi et al., 2024). However, the obtained *in vitro* data suggesting lower analytical detection thresholds, and thus potentially higher analytical sensitivity, provide grounds to expect its effectiveness in an area in which RUTs have so far not proved definitively useful, namely “sweeping” in the so-called gray zone of breath test results after eradication (Huang et al., 1998). Against this background, the low reaction threshold of the Helibac RUT is promising, but the clinically most important issue will be to verify whether greater analytical sensitivity does not increase the number of equivocal or false-positive readings in actual biopsy specimens.

From a regulatory perspective, the study serves as a preliminary assessment of the performance of diagnostic device within the legal frames of IVDR. IVDR defines “analytical performance” as the ability of a device to correctly detect or measure a given analyte, and “performance evaluation” as an assessment of data concerning scientific validity, analytical performance, and, where applicable, clinical performance of the device (European Parliament and Council of the European Union, 2017). From the perspective of IVDR, the adopted endpoints are consistent with the nature of a qualitative diagnostic device.

The analytical advantages observed in the present study should be interpreted within the broader context of rapid urease test development. Previous investigations have demonstrated that assay performance depends not only on urease activity itself but also on physical characteristics of the reaction environment, including diffusion distance, matrix composition, moisture retention, and specimen contact area (Laine et al., 1996; Niv et al., 1998; Vaira et al., 2010; Uotani and Graham, 2015). Studies evaluating optimized dry-format rapid urease tests, including Pronto Dry and ultrafast urease assays, have shown that modifications of matrix architecture may significantly shorten time-to-result and improve early-readout sensitivity while maintaining high specificity (Morio et al., 2004; Perna et al., 2005; Vaira et al., 2010). The rapid response observed with the Helibac RUT may therefore reflect improved organization of the reaction system rather than differences in the underlying biochemical mechanism. When interpreting these findings, it should be emphasized that the present work represents an analytical performance study conducted under controlled laboratory conditions. Although the results indicate favorable analytical characteristics, including rapid reaction kinetics and low detection thresholds, confirmation of these observations in prospective clinical studies using gastric biopsy specimens remains necessary. Future investigations should assess diagnostic sensitivity, specificity, predictive values, interobserver reproducibility, and agreement with histopathology, culture, and molecular reference methods (Vaira et al., 2000; Ricci et al., 2007; Uotani and Graham, 2015; Ansari and Yamaoka, 2022; Urgessa et al., 2023).

## Study limitations

Several limitations should be acknowledged. First, this study represents an analytical laboratory evaluation and does not constitute a clinical performance study. The experiments were conducted using standardized bacterial suspensions and purified urease rather than clinical gastric biopsy specimens. Consequently, the results do not account for biological variability associated with tissue sampling, bacterial distribution within the gastric mucosa, bleeding, proton pump inhibitor exposure, or other clinical factors known to affect rapid urease test performance. Second, experiments were performed primarily at 21 °C under controlled laboratory conditions. Finally, the study design does not allow identification of the individual contribution of each structural component of the cassette system to the observed analytical performance. Future investigations should therefore evaluate diagnostic accuracy, reproducibility, and clinical utility in real-world endoscopic settings.

## Conclusion

The Helibac RUT demonstrated favorable analytical performance characteristics under controlled laboratory conditions, including lower analytical detection thresholds and faster reaction kinetics compared with the evaluated comparator assays. These findings support further clinical validation studies and suggest that the cassette-based design may represent a promising approach for optimization of rapid urease testing.

Future studies should include prospective clinical performance evaluation using gastric biopsy specimens collected during routine upper gastrointestinal endoscopy. Such studies should assess diagnostic sensitivity, specificity, predictive values, and agreement with histopathology, culture, and molecular reference methods

## Data Availability

The raw data supporting the conclusions of this article will be made available by the authors, without undue reservation.
