# Supplementary material for: Analytical performance evaluation of the Helibac rapid urease test for detection of Helicobacter pylori urease activity under controlled laboratory conditions
Source: Front Microbiol. 2026 Jul 28;17:1889913. doi: 10.3389/fmicb.2026.1889913 (PMC13457363; doi:10.3389/fmicb.2026.1889913)
Supplement: Supplementary file 1 [file Data_Sheet_1.docx]

**Supplement**

**Table 1. Physical forms of the reaction system in rapid urease tests (RUTs) used in clinical and scientific practice**

| **Test** | **Physical form of the reaction system** |
| --- | --- |
| CLO test | Well in a plastic slide filled with agar gel containing urea, phenol red, buffers, and a bacteriostatic agent; the biopsy specimen is placed in the gel [19] |
| Hpfast | Well in the test device filled with agar gel containing urea, bromothymol blue, and methyl red; the biopsy specimen is placed in the gel [20] |
| PyloriTek | Reagent strip; urea is contained in the pad, and ammonia produced in the presence of urease diffuses through the membrane into the layer with the pH indicator [21] |
| Pronto Dry | Dry test with a reaction field and an external color-reading window/eyelet; the biopsy specimen is placed on the reactive area of the test [22] |
| Endosc-HP | Two-well cartridge; the reagents are contained in a tablet comprising urea, phenol red, and buffer salts, activated with a separate buffer [23] |
| Ad hoc solutions | Test tube or vessel with a solution of urea and a pH indicator; the biopsy specimen is immersed directly in the reaction solution [24] |

**Table 2. The speed of the urease reaction of the Helibac RUT, CLO test, ENDOSC-Hp and BIOHIT tests depending on the number of *H. pylori* cells at temperature 21°C.**

| Time of the urease reaction | **The number of *H. pylori* cells** | | | | | | | | |
| --- | --- | --- | --- | --- | --- | --- | --- | --- | --- |
|  | 10^7^ | 5,0x10^6^ | 2,5x10^6^ | 1,2x10^6^ | 6,0x10^5^ | 3,0x10^5^ | 1,5x10^5^ | 7,5x10^4^ | 3,7x10^4^ |
| **The Helibac RUT test**  1 min  5 min  10 min  30 min  60 min | ++++  ++++  ++++  ++++  ++++ | ++++  ++++  ++++  ++++  ++++ | ++++  ++++  ++++  ++++  ++++ | +++  +++  ++++  ++++  ++++ | +++  +++  ++++  ++++  ++++ | +  +++  ++++  ++++  ++++ | -  -  +++  +++  +++ | -  -  +  ++  +++ | -  -  -  +  ++ |
| **CLO test**  1 min  5 min  10 min  30 min  60 min | -  ++  +++  ++++  ++++ | -  ++  ++  +++  ++++ | -  +  ++  +++  +++ | -  -  +  ++  +++ | -  -  -  +  ++ | -  -  -  -  - | -  -  -  -  - | -  -  -  -  - | -  -  -  -  - |
| **BIOHIT**  1 min  5 min  10 min  30 min  60 min | -  ++  +++  +++  ++++ | -  +  ++  ++  +++ | -  -  +  +  ++ | -  -  -  +  ++ | -  -  -  -  ++ | -  -  -  -  - | -  -  -  -  - | -  -  -  -  - | -  -  -  -  - |
| **ENDOSC-***Hp*  1 min  5 min  10 min  30 min  60 min | -  +  ++  +++  ++++ | -  -  +  ++  +++ | -  -  +  +  ++ | -  -  -  -  ++ | -  -  -  -  + | -  -  -  -  - | -  -  -  -  - | -  -  -  -  - | -  -  -  -  - |

Reaction : ++++ crimson; +++ amaranth; ++ dark pink; + light pink; no color change

**Table 3. Urease reaction rate at temperature 21°C with SIGMA urease and detection threshold**

| Urease units transferred onto the test | | | | | | | |
| --- | --- | --- | --- | --- | --- | --- | --- |
| The Helibac RUT test | | | | | | | |
| Time | 0.10 | 0,05 | 0,025 | 0.012 | 0.006 | 0.003 | 0.0015 |
| 1 min  5 min  10 min  30 min  60 min | ++++  ++++  ++++  ++++  ++++ | +++  ++++  ++++  ++++  ++++ | +  +++  ++++  ++++  ++++ | +  ++  +++  ++++  ++++ | +  ++  +++  +++  +++ | -  -  +  ++  ++ | -  -  -  +  + |
| CLO test | | | | | | | |
| Time | 0.10 | 0,05 | 0,025 | 0.012 | 0.006 | 0.003 | 0.0015 |
| 1 min  5 min  10 min  30 min  60 min | -  ++  ++  +++  ++++ | -  +  ++  ++  +++ | -  -  +  ++  ++ | -  -  -  +  + | -  -  -  -  + | -  -  -  -  - | -  -  -  -  - |
| BIOHIT test | | | | | | | |
| Time | 0.10 | 0,05 | 0,025 | 0.012 | 0.006 | 0.003 | 0.0015 |
| 1 min  5 min  10 min  30 min  60 min | -  -  -  -  - | -  -  -  -  - | -  -  -  -  - | -  -  -  -  - | -  -  -  -  - | -  -  -  -  - | -  -  -  -  - |
| Endosc- *Hp* Test | | | | | | | |
| Time | 0.10 | 0,05 | 0,025 | 0.012 | 0.006 | 0.003 | 0.0015 |
| 1 min  5 min  10 min  30 min  60 min | -  -  -  -  - | -  -  -  -  - | -  -  -  -  - | -  -  -  -  - | -  -  -  -  - | -  -  -  -  - | -  -  -  -  - |

reaction : ++++ crimson; +++ amaranth; ++ dark pink; + light pink; no color change

**Table 4. Positive reaction in 21°C with definite quantity of urease in 5 microliters of distilled water transferred onto urease tests produced in Poland.**

| Time | Number of urease units | RUT-s | | |
| --- | --- | --- | --- | --- |
|  |  | The Helibac RUT | Manufacturer no 1. | Manufacturer no 2. |
| 5 minutes | 0,006  0,003  0,0015 | +++++  +++  + |  |  |
| 15 minutes | 0,006  0,003  0,0015 | +++++  ++++  ++ |  |  |
| 30 minutes | 0,006  0,003  0,0015 | +++++  +++++  +++ | +++++  +++  + | ++ |
| 60 minutes | 0,006  0,003  0,0015 | +++++  +++++  ++++ | +++++  ++  + | + |

Note: In Table 4, the number of "+" symbols represents the number of positive replicate tests (n = 5) and not the intensity of color development.

**Table 5. Mean time to the first positive reaction, standard deviation, and 95% confidence intervals for the analyzed rapid urease tests.**

| **Test** | **Mean Time to First Positive Reaction (min)** | **SD** | **95% CI (min)** |
| --- | --- | --- | --- |
| Helibac RUT | 17.9 | 5.2 | 16.42–19.38 |
| CLO Test | 39.0 | 11.5 | 35.73–42.27 |
| BIOHIT | 85.6 | 24.0 | 78.78–92.42 |
| ENDOSC-Hp | 91.1 | 26.5 | 83.57–98.63 |
